# Time-Domain and Monostatic-like Frequency-Domain Methods for Bistatic SAR Simulation

**DOI:** 10.3390/s21155012

**Published:** 2021-07-23

**Authors:** Gerardo Di Martino, Antonio Iodice, Antonio Natale, Daniele Riccio

**Affiliations:** 1 Dipartimento di Ingegneria Elettrica e delle Tecnologie dell’Informazione, Università di Napoli Federico II, 80125 Napoli, Italy; daniele.riccio@unina.it; 2 Istituto per il Rilevamento Elettromagnetico dell’Ambiente (IREA), Consiglio Nazionale delle Ricerche (CNR), 80124 Napoli, Italy; natale.a@irea.cnr.it

**Keywords:** bistatic SAR, SAR simulation, SAR processing

## Abstract

In recent years, an increasing interest has been devoted to bistatic SAR configurations, which can be effectively used to improve system performance and/or to increase the amount of physical information retrievable from the observed scene. Within this context, the availability of simulation tools is of paramount importance, for both mission planning and processing algorithm verification and testing. In this paper, a time domain simulator useful to obtain the point-spread function and the raw signal for the generic bistatic SAR configuration is presented. Moreover, we focus on the case of two bistatic configurations, which are of considerable interest in actual SAR applications, i.e., the translational invariant SAR and the one-stationary SAR acquisition geometries, for which we obtain meaningful expressions of the Transfer Functions. In particular, these expressions are formally equal to those obtained for the monostatic SAR configuration, so that the already available monostatic simulator can be easily adapted to these bistatic cases. The point-target raw signals obtained using the (exact) time domain simulator and the (approximated) frequency domain one are compared, with special attention to acquisition geometries that may be of practical interest in Formation-Flying SAR applications. Results show that the phase difference between raw signals simulated with the two approaches is, in all cases, smaller (and often much smaller) than about 10 degrees, except that at the very edge of the raw signals, where however, it does not exceed about 50 degrees.

## 1. Introduction

Until now, Synthetic Aperture Radar (SAR) missions have been designed mostly to work in monostatic configuration. However, in recent years, an increasing interest has been devoted to bistatic and multistatic SAR acquisition geometries [1,2,3,4,5,6]. Indeed, the presence of a spatial separation between receiver and transmitter platforms can be effectively exploited to improve system performance and to widen the range of retrievable physical information from the acquired data [7,8,9,10,11,12,13,14]. For instance, in the framework of Formation Flying SAR (FF-SAR), specific acquisition configurations can be considered in order to improve the signal-to-noise ratio (SNR), the spatial resolution, and the range coverage [7,8,9,10]. Moreover, more general bistatic/multistatic configurations grant an increased number of degrees of freedom with respect to the monostatic one in terms of acquisition geometry (no longer restricted to the backscattering case) that could be used to increase the retrievable information about the observed scene, also thanks to the availability of appropriate bistatic scattering models [11,12,13,14].

SAR data simulation can play a key role in the investigation of the special principles and properties of bistatic image formation. Indeed, simulation is of paramount importance in the mission planning phase, in order to define the most suitable acquisition geometries in terms of extension and position of the imaged scene, attainable SNR and resolutions, and so on. Moreover, the availability of simulated raw data is a prerequisite for the development and testing of SAR processing techniques. However, while several contributions in the scientific literature have addressed the problem of SAR raw signal simulation for a wide range of imaged scenes, sensing platforms, and acquisition modes [15,16,17,18,19,20], only few contributions focused on the bistatic case are available [21,22,23,24]. Most of them consider specific acquisition geometries, such as the translational invariant (TI) [21,22], where the transmitter and the receiver share the same velocity vector (i.e., they move along parallel orbits with the same velocity), and the one-stationary bistatic configuration [24], where the receiver is fixed and placed close to the Earth surface. In these special cases, simplified formulations of the system impulse response and transfer function (TF) can be obtained and, consequently, computationally efficient processing schemes can be devised.

As a matter of fact, these acquisition geometries are of special interest in practical scenarios. The TI configuration well describes the basic configuration of an FF-SAR, where the flying paths of two (or more) platforms are jointly planned to guarantee the best working conditions for attaining specific tasks (e.g., SNR enhancement, interferometry applications, wide-swath imaging) [7,8,9,10]. As for the one-stationary configuration, it can be fruitfully used to exploit the signal transmitted by either orbiting satellites (space-surface configuration) or ad hoc planned airborne platforms [24]. The most general case in which two (or more) platforms follow arbitrary tracks may be of interest whenever the exploitation of already orbiting spaceborne SAR systems is considered: however, even if this scenario will certainly gain increasing attention in the next few years, it is, at present, of less practical interest.

Within the general framework of bistatic raw signal simulation, the contribution of the present paper is twofold. First, we present a SAR simulator working in the time domain (TD) able to obtain the point-spread function (PSF) and the raw signal for generic bistatic configurations. This is accomplished defining an appropriate *look function* of the bistatic system, i.e., the function describing the time interval in which a point target is simultaneously seen from both the transmitter and the receiver. The TD analysis and PSF derivation are presented in Section 2. The second contribution, which represents the major result of this work, is relevant to the TI and one-stationary acquisition geometries. In particular, a frequency-domain simulation scheme is proposed, based on the evaluation of the TFs for the two considered bistatic configurations. The obtained formulation is formally identical to the one obtained in [15,16,17,18,19,20,25] for the monostatic case, provided that the expressions of some of the involved parameters are appropriately modified. This allows to devise computationally efficient processing schemes, suitable for extended scene simulation and working in the frequency domain [25], very similar to those employed for monostatic SAR simulation in [15,16,17,18,19,20]. Accordingly, the available monostatic simulator can be easily adapted to the bistatic case. The frequency domain simulation scheme for the TI and one-stationary bistatic SAR configurations are discussed in Section 3. The results obtained via TD and frequency-domain simulation are compared and discussed in Section 4, with special attention to acquisition geometries that are of practical interest in FF-SAR applications. Relevant conclusions of the paper are drawn in Section 5.

## 2. Time-Domain Bistatic SAR Simulation

The developed time-domain (TD) simulator is able to evaluate the two-dimensional pulse response for a generic bistatic SAR configuration, starting from the input sensors’ parameters, i.e., velocities, trajectories, bandwidths, antenna’s characteristics. Preliminary concepts of the presented approach have been presented in [26]. Here, the approach is fully described and extended by including possible squint angles for both transmitter and receiver.

**Figure 1:**
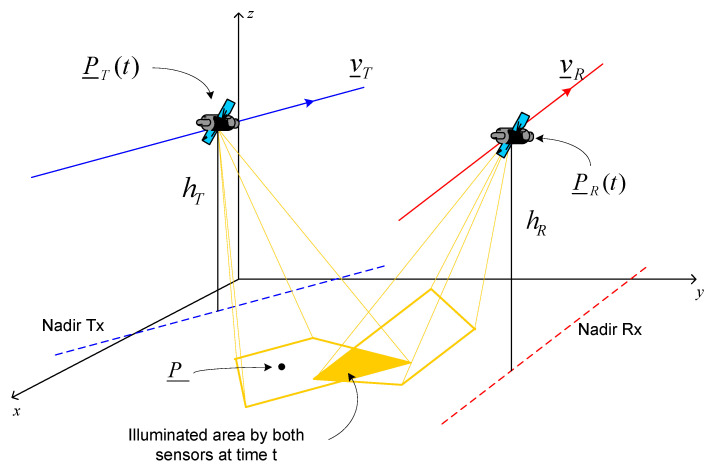
Geometry of the bistatic SAR configuration.

Let *P_T_*(*t*) and *P_R_*(*t*) be the trajectories of the transmitter and receiver, respectively, which are assumed to move of uniform rectilinear motion (a reasonable approximation for a very short section of orbit), so that we can write:

(1) $${\underline{P}}_{T}\left(t\right)=\left(x_{T}\left(t\right),y_{T}\left(t\right),h_{T}\right)=\{\begin{array}{l} x_{T}\left(t_{0}\right)+v_{T}v_{xT}\left(t-t_{0}\right)\\ y_{T}\left(t_{0}\right)+v_{T}v_{yT}\left(t-t_{0}\right)\\ h_{T} \end{array}$$

and

(2) $${\underline{P}}_{R}\left(t\right)=\left(x_{R}\left(t\right),y_{R}\left(t\right),h_{R}\right)=\{\begin{array}{l} x_{R}\left(t_{0}\right)+v_{R}v_{xR}\left(t-t_{0}\right)\\ y_{R}\left(t_{0}\right)+v_{R}v_{yR}\left(t-t_{0}\right)\\ h_{R} \end{array}$$

where *t*_0_ is the starting observation time; ${\underline{v}}_{T}=v_{T}{\hat{v}}_{T}\;\mathrm{with}\;{\hat{v}}_{T}\equiv \left(v_{xT},v_{yT},0\right)$, and ${\underline{v}}_{R}=v_{R}{\hat{v}}_{R}\;\mathrm{with}\;{\hat{v}}_{R}\equiv \left(v_{xR},v_{yR},0\right)$, are the transmitter’s and receiver’s velocity vectors, respectively; and *h_T_* and *h_R_* are their heights. The geometry of the considered bistatic SAR configuration is shown in Figure 1. The transmitting antenna along-track and cross-track sizes are *L_T_* and *W_T_*, respectively. For the receiving antenna, they are *L_R_* and *W_R_*.

**Figure 2:**
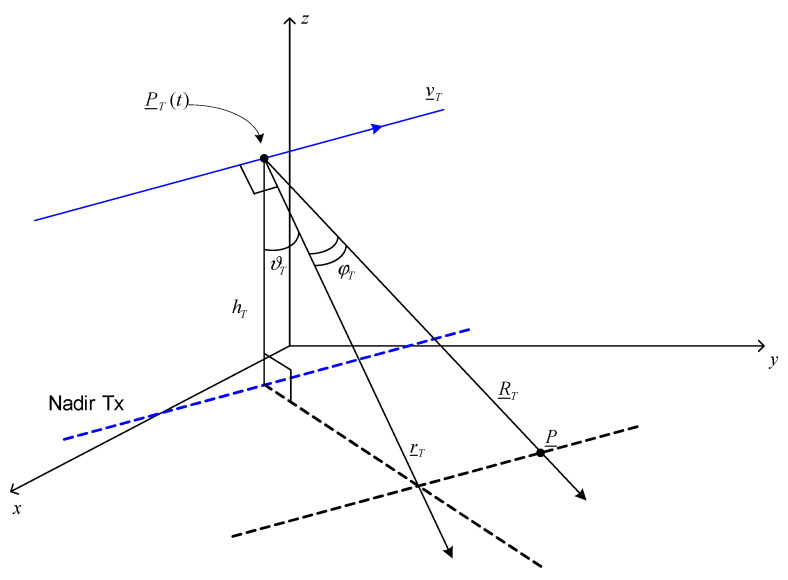
Transmitter’s geometry.

Let us suppose that $\underline{P}=\left(x,y,z\right)$ is the only ground target present on the scene, generating a non-negligible electromagnetic response at the receiver side. In order to obtain the PSF, we are interested in the evaluation of the transmitter-to-target distance vector at the time *t*, that is ${\underline{R}}_{T}\left({\underline{P}}_{T},\underline{P},t\right)=\underline{P}-{\underline{P}}_{T}\left(t\right)$, which can be decomposed in its (local) azimuth and range components:

(3) $${\underline{R}}_{T}\left({\underline{P}}_{T},\underline{P},t\right)=R_{T}{\hat{R}}_{T}=R_{T}\left({\hat{r}}_{T}\cos\phi_{T}+{\hat{v}}_{T}\sin\phi_{T}\right)$$

where ${\hat{r}}_{T}=\left(v_{yT}\sin\vartheta_{T},-v_{xT}\sin\vartheta_{T},-\cos\vartheta_{T}\right)$ is the zero-Doppler direction for the transmitter at the time *t*, while $\phi_{T}$ and $\vartheta_{T}$ are respectively the azimuth angle and the elevation angle according to which the point P is seen from the transmitter. Details about the transmitter’s geometry are provided in Figure 2.

The angles $\phi_{T}$ and $\vartheta_{T}$ can be related to the coordinates of the transmitter and of the target point *P*:

(4) $$\phi_{T}\left({\underline{P}}_{T},\underline{P},t\right)=\sin^{-1}\left(\frac{{\underline{R}}_{T}\cdot {\hat{v}}_{T}}{R_{T}}\right)=\sin^{-1}\left(\frac{\left(x-x_{T}\right)v_{xT}+\left(y-y_{T}\right)v_{yT}}{\sqrt{{\left(x-x_{T}\right)}^{2}+{\left(y-y_{T}\right)}^{2}+{\left(h_{T}-z\right)}^{2}}}\right)$$

and

(5) $$\vartheta_{T}\left({\underline{P}}_{T},\underline{P},t\right)=\cos^{-1}\left(\frac{h_{T}}{R_{T}\cos\phi_{T}}\right)$$

The knowledge of antenna’s azimuth and range angular apertures, which can be assumed equal to $\Delta \phi_{T}≅\lambda /L_{T}$ and $\Delta \vartheta_{T}≅\lambda /W_{T}$, together with the analytical expression in (4) and (5) and the knowledge of the transmitter look angle $\vartheta_{0T}$ and squint angle $\phi_{0T}$, allow us to know at any time if the scattering point *P* is illuminated by the transmitter. This can be expressed introducing a transmitter illumination function:

(6) $$L_{T}\left({\underline{P}}_{T},\Delta \phi_{T},\Delta \vartheta_{T},\vartheta_{0T},\phi_{0T},\underline{P},t\right)=\mathrm{rect}\left(\frac{\vartheta_{T}\left({\underline{P}}_{T},\underline{P},t\right)-\vartheta_{0T}}{\Delta \vartheta_{T}}\right)\mathrm{rect}\left(\frac{\phi_{T}\left({\underline{P}}_{T},\underline{P},t\right)-\phi_{0T}}{\Delta \phi_{T}}\right)$$

where rect(x) is the rectangular window function, equal to 1 if |*x*| ≤ 1/2 and to 0 otherwise. Similarly, it is also possible to define a receiver illumination function:

(7) $$L_{R}\left({\underline{P}}_{R},\Delta \phi_{R},\Delta \vartheta_{R},\vartheta_{0R},\phi_{0R},\underline{P},t\right)=\mathrm{rect}\left(\frac{\vartheta_{R}\left({\underline{P}}_{R},\underline{P},t\right)-\vartheta_{0R}}{\Delta \vartheta_{R}}\right)\mathrm{rect}\left(\frac{\phi_{R}\left({\underline{P}}_{R},\underline{P},t\right)-\phi_{0R}}{\Delta \phi_{R}}\right)$$

The product of (6) and (7) represents the look function of the bistatic system, L(·), i.e., the function describing the time interval in which the point *P* is simultaneously seen from both the transmitter and the receiver.

Thanks to the definition of this look function, the evaluation of the bistatic PSF can be easily completed. Indeed, if $f_{Tx}\left(t-t_{n}\right)$ represents the *n*-th pulse (e.g., the usual chirp), which is transmitted at time $t_{n}$, and if we denote the transmitting and receiving antenna’s patterns with $W_{T}\left(\cdot \right)$ and $W_{R}\left(\cdot \right)$, respectively, then the bistatic PSF is expressed by:

(8) $$f_{Rx}\left(\underline{P},{\underline{P}}_{T},{\underline{P}}_{R},t,t_{n}\right)=f_{Tx}\left(t-t_{n}-\frac{R_{T}+R_{R}}{c}\right)W_{T}\left(\underline{P},{\underline{P}}_{T},t_{n}\right)W_{R}\left(\underline{P},{\underline{P}}_{R},t_{n}\right)L\left(\underline{P},{\underline{P}}_{T},{\underline{P}}_{R},t_{n}\right)$$

where *c* is the speed of light and (1)–(7) are evaluated for *t* = *t_n_* (i.e., the usual stop-and-start approximation [25] is made). In (8), we have ignored a multiplicative constant, depending on transmitted power and antenna gain.

Unlike the monostatic configuration, in which the range-azimuth plane corresponds to the SAR image plane, here a local range-azimuth plane for both the transmitter and receiver side is defined. However, none of these local planes correspond to the bistatic image plane, that is rather defined in the so-called slow-time/fast-time reference system: the slow time is the time history, i.e., $t_{S}=t_{n}$, while the fast time represents the time elapsed from each pulse transmission, i.e., $t_{F}=t-t_{n}$.

Accordingly, the bistatic pulse response (8) can be rewritten as:

(9) $$f_{Rx}\left(\underline{P},{\underline{P}}_{T},{\underline{P}}_{R},t_{S},t_{F}\right)=f_{Tx}\left(t_{F}-\frac{R_{T}+R_{R}}{c}\right)W_{T}\left(\underline{P},{\underline{P}}_{T},t_{S}\right)W_{R}\left(\underline{P},{\underline{P}}_{R},t_{S}\right)L\left(\underline{P},{\underline{P}}_{T},{\underline{P}}_{R},t_{S}\right)$$

that is usually time varying with respect to both the slow time and the fast time.

Such a point-spread function can be used to express the bistatic SAR raw signal h(·), through the superposition integral:

(10) $$h\left({\underline{P}}_{T},{\underline{P}}_{R},t_{S},t_{F}\right)=\int \gamma \left(\underline{P}\right)f_{Rx}\left(\underline{P},{\underline{P}}_{T},{\underline{P}}_{R},t_{S},t_{F}\right)d\underline{P}$$

where $\gamma \left(\underline{P}\right)$ is the bistatic reflectivity function, that can be computed by using, e.g., the model of [14] for natural terrain or sea surface, and the model of [27,28] for buildings. However, implementation of the integral in (10) in time domain is very time consuming, so that extended-scene raw signal simulators should be implemented in frequency domain. 

## 3. Frequency-Domain Bistatic SAR Simulation

Efficient frequency-domain bistatic SAR simulation schemes can be achieved in particular configurations. We here consider the TI and the one-stationary bistatic SAR configurations and show that in these cases the system TF can be expresses in a form analogous to the one of a monostatic SAR [15,16,17,18,19,20], so that efficient frequency-domain SAR raw signal simulators available for the monostatic SAR [15,16,17,18,19,20] can be easily adapted to the bistatic case. 

### 3.1. Translational Invariant Configuration

**Figure 3:**
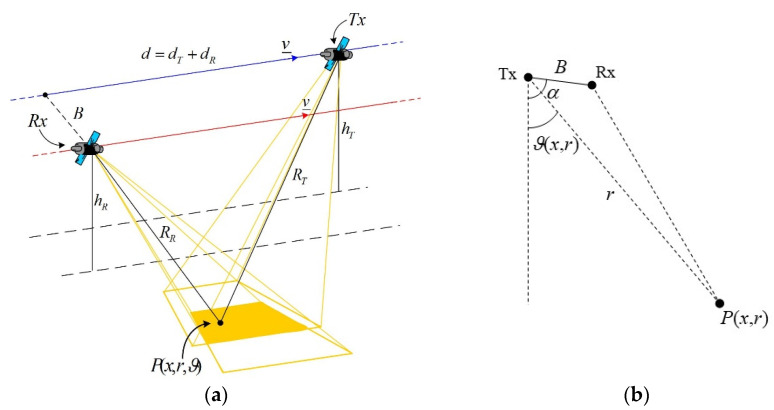
Geometry of the TI bistatic SAR configuration: (**a**) 3D view; (**b**) cross-track plane view.

In this configuration, we have $v_{T}=v_{R}=v$, ${\hat{v}}_{T}={\hat{v}}_{R}=\hat{v}\equiv \left(1,0,0\right)$, ${\underline{v}}_{T}={\underline{v}}_{R}=\underline{v}$, so that:

(11) $${\underline{P}}_{T}\left(t_{n}\right)=\left(x_{T}\left(t_{n}\right),y_{T},h_{T}\right)=\{\begin{array}{l} vt_{n}+d_{T}=x^{\prime }+d_{T}\\ 0\\ h_{T} \end{array}$$

and

(12) $${\underline{P}}_{R}\left(t_{n}\right)=\left(x_{R}\left(t_{n}\right),y_{R},h_{R}\right)=\{\begin{array}{l} vt_{n}-d_{R}=x^{\prime }-d_{R}\\ B\sin\alpha \\ h_{T}-Bcos\alpha \end{array}$$

where $d=d_{T}+d_{R}$ is the along-track baseline, B is the cross-track baseline and α is the cross-track baseline angle, see Figure 3. We consider a stripmap acquisition mode, and we assume that the antennas are pointed in such a way as to illuminate a common area on the ground whose center has an azimuth coordinate *x*′ = *vt_n_* (i.e., transmitting and receiving antennas are both squinted), so that the illumination function is:

(13) $$L\left(\underline{P},{\underline{P}}_{T},{\underline{P}}_{R},t_{n}\right)=L\left(r,x^{\prime }-x\right)=\mathrm{rect}\left(\frac{r-r_{0}}{S_{r}}\right)\mathrm{rect}\left(\frac{x-x^{\prime }}{X}\right)$$

where $r=\sqrt{y^{2}+{\left(h_{T}-z\right)}^{2}}$ is the slant range (from the transmitter) of the generic ground point, *r*_0_ is the slant range of the center of the illuminated area, and *S_r_* and *X* are the slant range and azimuth sizes of the common illuminated area, respectively, so that *X* is the smaller between the transmitter’s, *X_T_*, and the receiver’s, *X_R_*, azimuth antenna footprints. The bistatic PSF of (8) is then expressed as:

(14) $$f_{Rx}\left(x^{\prime },x,t-t_{n},r\right)=f_{Tx}\left(t-t_{n}-\frac{R_{T}+R_{R}}{c}\right)W_{T}\left(\frac{x^{\prime }-x}{X_{T}}\right)W_{R}\left(\frac{x^{\prime }-x}{X_{R}}\right)L\left(r,x^{\prime }-x\right)$$

and the bistatic SAR raw signal as:

(15) $$h\left(x^{\prime },t-t_{n}\right)=\iint \gamma \left(x,r\right)f_{Rx}\left(x^{\prime },x,t-t_{n},r\right)dxdr$$

In view of (11) and (12), the target-to-sensors distances $R_{T}\;\mathrm{and}\;R_{R}$ can be written as:

(16) $$\begin{array}{l} R_{T}=\sqrt{r^{2}+{\left(x^{\prime }+d_{T}-x\right)}^{2}}=\\ =\sqrt{r^{2}+d_{T}^{2}+2d_{T}\left(x^{\prime }-x\right)+{\left(x^{\prime }-x\right)}^{2}}≅\\ ≅\frac{r}{\cos\psi_{T}}+\sin\psi_{T}\left(x^{\prime }-x\right)+\cos^{3}\psi_{T}\frac{{\left(x^{\prime }-x\right)}^{2}}{2r} \end{array}$$

(17) $$\begin{array}{l} R_{R}=\sqrt{r^{2}-2rB\cos\left(\alpha -\vartheta \right)+B^{2}+{\left(x^{\prime }-d_{R}-x\right)}^{2}}=\\ =\sqrt{r^{2}+d_{R}^{2}-2rB\cos\left(\alpha -\vartheta \right)+B^{2}-2d_{R}\left(x^{\prime }-x\right)+{\left(x^{\prime }-x\right)}^{2}}≅\\ ≅\frac{r}{\cos\psi_{R}}-\sin\psi_{R}\left(x^{\prime }-x\right)+\cos^{3}\psi_{R}\frac{{\left(x^{\prime }-x\right)}^{2}}{2r}+\\ -\cos\psi_{R}B\cos\left(\alpha -\vartheta \right)+\cos\psi_{R}\frac{B^{2}}{2r}\left[1-\cos^{2}\psi_{R}\cos^{2}\left(\alpha -\vartheta \right)\right] \end{array}$$

where the elevation angle $\vartheta =\vartheta_{T}\left(x,r\right)$ describes the scene height profile, and

(18) $$\cos\psi_{T}=\frac{r}{\sqrt{r^{2}+d_{T}^{2}}},\;\;\;\;\sin\psi_{T}=\frac{d_{T}}{\sqrt{r^{2}+d_{T}^{2}}},\;\;\;\;\;\cos\psi_{R}=\frac{r}{\sqrt{r^{2}+d_{R}^{2}}},\;\;\;\;\sin\psi_{R}=\frac{d_{R}}{\sqrt{r^{2}+d_{R}^{2}}}.\;\;$$

Therefore,

(19) $$R_{T}+R_{R}≅\beta \left(r\right)r+\Delta r_{c}\left(r,\vartheta \right)+\Delta R\left(x^{\prime }-x,r\right)$$

with

(20) $$\beta \left(r\right)=\frac{\cos\psi_{T}+\cos\psi_{R}}{\cos\psi_{T}\cos\psi_{R}}$$

(21) $$\Delta r_{c}\left(r,\vartheta \right)=-\cos\psi_{R}B\cos\left(\alpha -\vartheta \right)+\cos\psi_{R}\frac{B^{2}}{2r}\left[1-\cos^{2}\psi_{R}\cos^{2}\left(\alpha -\vartheta \right)\right]$$

(22) $$\Delta R\left(x^{\prime }-x,r\right)=(\sin\psi_{T}-\sin\psi_{R})\left(x\prime -x\right)+\frac{\left(\cos^{3}\psi_{T}+\cos^{3}\psi_{R}\right)}{2r}{\left(x^{\prime }-x\right)}^{2}$$

Note that in (16)–(22) we are assuming *X* << *r* and *B* << *r*, whereas no strict constraint is posed on *d*. By assuming that the transmitted pulse is an up-chirp with bandwidth Δf and duration τ, the PSF (after coherent demodulation of the raw signal) can be written as:

(23) $$\begin{array}{c} f_{Rx}\left(x\prime ,x,t-t_{n},r\right)=\exp\left[-j\frac{2\pi }{\lambda }\left(\beta r+\Delta r_{c}+\Delta R\right)\right]\\ \exp\left[j\frac{\pi \Delta f}{\tau }{\left(t-t_{n}-\frac{\beta r+\Delta r_{c}+\Delta R}{c}\right)}^{2}\right]\\ \mathrm{rect}\left[\frac{1}{\tau }\left(t-t_{n}-\frac{\beta r+\Delta r_{c}+\Delta R}{c}\right)\right]\mathrm{rect}\left(\frac{r-r_{0}}{S_{r}}\right)\mathrm{rect}\left(\frac{x^{\prime }-x}{X}\right)W_{T}\left(\frac{x^{\prime }-x}{X_{T}}\right)W_{R}\left(\frac{x^{\prime }-x}{X_{R}}\right) \end{array}$$

Finally, by letting $r^{\prime }=\frac{c}{\beta_{0}}\left(t-t_{n}\right)$, with $\beta_{0}=\beta \left(r_{0}\right)$, the raw signal in (15) can be expressed as:

(24) $$h\left(x^{\prime },r^{\prime }\right)=\iint \tilde{\gamma }\left(x,r\right)g\left(x^{\prime }-x,\;r^{\prime }-r;r\right)dxdr$$

where

(25) $$\tilde{\gamma }\left(x,r\right)=\gamma \left(x,r\right)\exp\left\{-j\frac{2\pi }{\lambda }\left[\beta \left(r\right)r+\Delta r_{c}\left(r,\vartheta \left(x,r\right)\right)\right]\right\}\mathrm{rect}\left(\frac{r-r_{0}}{S_{r}}\right)$$

and

(26) $$\begin{array}{l} g\left(x^{\prime }-x,\;r^{\prime }-r;r\right)≅\exp\left[j\xi_{DC}\left(x^{\prime }-x\right)-ja{\left(x^{\prime }-x\right)}^{2}\right]\\ \exp\left[jb{\left(r^{\prime }-r-\left(\beta -\beta_{0}\right)r-\frac{2\pi \Delta r_{cf}-\lambda \xi_{DC}\left(x^{\prime }-x\right)+\lambda a{\left(x^{\prime }-x\right)}^{2}}{2\pi \beta_{0}}\right)}^{2}\right]\\ \mathrm{rect}\left[\frac{\beta_{0}}{c\tau }\left(r^{\prime }-r-\left(\beta -\beta_{0}\right)r-\frac{2\pi \Delta r_{cf}-\lambda \xi_{DC}\left(x^{\prime }-x\right)+\lambda a{\left(x^{\prime }-x\right)}^{2}}{2\pi \beta_{0}}\right)\right]W\left(\frac{x^{\prime }-x}{X}\right) \end{array}$$

with

(27) $$\xi_{DC}=-\frac{2\pi }{\lambda }(\sin\psi_{T}-\sin\psi_{R}),\;a=\frac{\pi }{\lambda r}(\cos^{3}\psi_{T}+\cos^{3}\psi_{R}),\;b=\frac{\beta_{0}^{2}\pi }{\lambda }\frac{\Delta f/f}{c\tau },$$

(28) $$\Delta r_{cf}=\Delta r_{c}\left(r,\vartheta_{flat}\left(r\right)\right)\;\;\mathrm{and}\;W\left(\frac{x^{\prime }-x}{X}\right)=\mathrm{rect}\left(\frac{x^{\prime }-x}{X}\right)W_{T}\left(\frac{x^{\prime }-x}{X_{T}}\right)W_{R}\left(\frac{x^{\prime }-x}{X_{R}}\right).$$

We explicitly note that $\xi_{DC}$, a and $\Delta r_{cf}$ are *r*-dependent, while *b* is a constant. In addition, we stress that in the argument of the exponential in (25) the exact value of the elevation angle ϑ(x,r) is used. Conversely, in (28), i.e., in the argument of the exponential in (26), the elevation angle ϑ(x,r) is approximated by the elevation angle $\vartheta_{flat}\left(r\right)$ corresponding to flat earth, to remove the *x*-dependence: this is acceptable, since *b* << (2*π*/*λ*)^2^, so that the approximation error on $\Delta r_{cf}^{2}$ multiplied by *b* is much smaller than unity. 

It is noteworthy that the TI bistatic SAR raw signal of (24)–(26) is of the same form of the monostatic SAR raw signal of [15,16,17,18,19,20,25], although with different expressions for the parameters $\xi_{DC}$, a and b. Therefore, the same asymptotic stationary-phase evaluation of its two-dimensional (2-D) Fourier Transform (FT) can be performed, thus obtaining:

(29) $$H\left(\xi ,\eta \right)=\iint \tilde{\gamma }\left(x,r\right)G\left(\xi ,\eta ;r\right)\exp\left(-j\xi x-j\eta r\right)dxdr,$$

where

(30) $$G\left(\xi ,\eta ;r\right)=\exp\left\{j\left[\frac{\xi^{2}}{4a\left(1+\frac{\eta \lambda }{2\pi \beta_{0}}\right)}-\frac{\xi \;\xi_{DC}}{2a}-\frac{\eta^{2}}{4b}+\frac{\;\eta \lambda }{2\pi \beta_{0}}\frac{\xi_{DC}^{2}}{4a}-\eta \left(\beta -\beta_{0}\right)r-\frac{\eta \;\Delta r_{cf}}{\beta_{0}}\right]\right\}W\left[\frac{\xi -\xi_{DC}}{2aX}\right]\mathrm{rect}\left[\frac{\eta }{2bc\tau /\beta_{0}}\right].$$

By using an approach similar to the one employed in [15,16,17,25], we can approximate G(ξ,η;r) as follows:

(31) $$G\left(\xi ,\eta ;r\right)≅G_{0}\left(\xi ,\eta \right)\exp\left[j\mu \left(\xi ,\eta \right)\left(r-r_{0}\right)\right],$$

where $G_{0}\left(\xi ,\eta \right)=G\left(\xi ,\eta ;r_{0}\right)$ and

(32) $$\mu \left(\xi ,\eta \right)={\frac{\partial }{\partial r}\Phi \left(\xi ,\eta ;r\right)|}_{r=r_{0}},$$

with

(33) $$\Phi \left(\xi ,\eta ;r\right)=\frac{\xi^{2}}{4a\left(1+\frac{\eta \lambda }{2\pi \beta_{0}}\right)}-\frac{\xi \;\xi_{DC}}{2a}+\frac{\;\eta \lambda }{2\pi \beta_{0}}\frac{\xi_{DC}^{2}}{4a}-\eta \left(\beta -\beta_{0}\right)r-\frac{\eta \;\Delta r_{cf}}{\beta_{0}}$$

By using (31) in (29) we get:

(34) $$H\left(\xi ,\eta \right)=\tilde{\Gamma }\left[\xi ,\eta -\mu \left(\xi ,\eta \right)\right]G_{0}\left(\xi ,\eta \right),$$

where $\tilde{\Gamma }\left[\xi ,\eta \right]$ is the 2-D FT of $\tilde{\gamma }\left(x,r\right)$. 

Accordingly, an extended-scene bistatic SAR raw signal simulation scheme similar to the monostatic one of [15,16,17,18,19,20] can be employed:

1. given the scene description in terms of Digital Elevation Model (DEM), complex dielectric constant and microscopic roughness of the surface, given the platform orbit data and given the SAR system parameters, the bistatic reflectivity map γ(x,r) is computed;
2. $\tilde{\gamma }\left(x,r\right)$ is computed from γ(x,r) by using (25);
3. the 2-D FT $\tilde{\Gamma }\left[\xi ,\eta \right]$ of $\tilde{\gamma }\left(x,r\right)$ is evaluated by using a Fast FT (FFT) algorithm;
4. interpolation in the Fourier domain is performed to obtain $\tilde{\Gamma }\left[\xi ,\eta -\mu \left(\xi ,\eta \right)\right]$ from $\tilde{\Gamma }\left[\xi ,\eta \right]$;
5. multiplication by $G_{0}\left(\xi ,\eta \right)$ is performed to obtain H(ξ,η) via (34);
6. the inverse 2-D FT $h\left(x^{\prime },r^{\prime }\right)$ of H(ξ,η) is evaluated by using a FFT algorithm.

Actually, in practice the 2-D FT of step 3 and the Fourier-domain interpolation of step 4 can be precisely and efficiently performed simultaneously by using a chirp-scaling algorithm, see [17,25]. An even simpler implementation can be used if *d* << *r* and the range swath *S_r_* is not very large, in which case we have:

(35) $$\mu \left(\xi ,\eta \right)≅\frac{\xi^{2}}{4a_{0}r_{0}}=\mu \left(\xi \right),\;\mathrm{with}\;a_{0}=a\left(r_{0}\right),$$

so that interpolation can be simply performed by multiplying the azimuth-transformed reflectivity by a linear (with respect to *r*) phase exponential before range-transforming it [17,25].

We finally explicitly note that in the particular case of *d* = 0 (i.e., no along-track baseline) we get the twin-SAR bistatic configuration considered in [26]. 

### 3.2. One-Stationary Configuration

**Figure 4:**
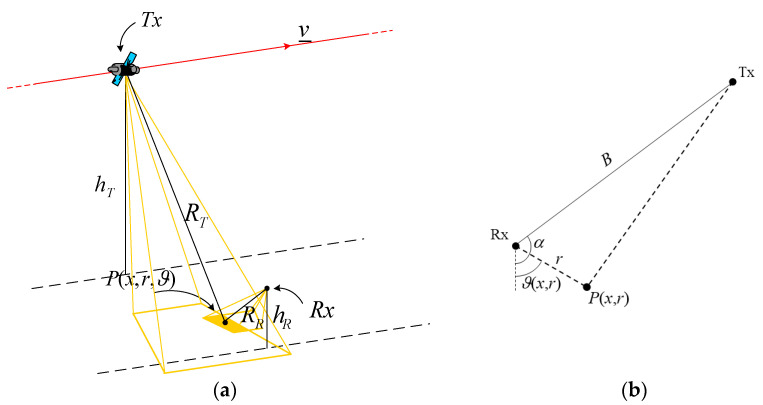
Geometry of the one-stationary bistatic SAR configuration: (**a**) 3D view; (**b**) cross-track plane view.

The one-stationary bistatic SAR is a system composed of a mobile transmitter and a ground-fixed receiver, as shown in Figure 4. The mobile transmitter may be either spaceborne (space-surface configuration, [26]) or airborne [24]. In addition, the receiver may be either in the forward-scattering region, as depicted in Figure 4, in which case *π*/2 < *α* < *π*, or in the back-scattering region, in which case *π* < *α* < 3*π*/2. The following formulation holds in both cases. 

In this configuration, we have $v_{T}=v\;$, ${\hat{v}}_{T}=\hat{v}\equiv \left(1,0,0\right)$, ${\underline{v}}_{R}=0$, so that:

(36) $${\underline{P}}_{T}\left(t_{n}\right)=\left(x_{T}\left(t_{n}\right),y_{T},h_{T}\right)=\{\begin{array}{l} vt_{n}=x^{\prime }\\ B\sin\alpha \\ h_{R}-B\cos\alpha \end{array}$$

and

(37) $${\underline{P}}_{R}=\left(x_{R},y_{R},h_{R}\right)=\{\begin{array}{l} 0\\ 0\\ h_{R} \end{array},$$

where B is the cross-track baseline and α is the cross-track baseline angle, see Figure 4. We consider a stripmap illumination mode for the transmitter, and we assume that the receiver antenna is pointed in such a way as to illuminate a portion of the transmitter’s range swath, so that the illumination function is:

(38) $$L\left(\underline{P},{\underline{P}}_{T},{\underline{P}}_{R},t_{n}\right)=L\left(x,x^{\prime },r\right)=\mathrm{rect}\left(\frac{r-r_{0}}{S_{r}}\right)\mathrm{rect}\left(\frac{x}{X_{R}}\right)\mathrm{rect}\left(\frac{x-x^{\prime }}{X_{T}}\right)$$

where $r=\sqrt{y^{2}+{\left(h_{R}-z\right)}^{2}}$ is the slant range (from the receiver) of the generic ground point, *r*_0_ is the slant range of the center of the receiver’s antenna footprint, and *S_r_* is the size of the receiver’s antenna footprint.

The bistatic raw signal is then expressed by (15) with:

(39) $$f_{Rx}\left(x^{\prime },x,t-t_{n},r\right)=f_{Tx}\left(t-t_{n}-\frac{R_{T}+R_{R}}{c}\right)W_{T}\left(\frac{x^{\prime }-x}{X_{T}}\right)W_{R}\left(\frac{x}{X_{R}}\right)L\left(x,x^{\prime },r\right).$$

In view of (36) and (37), and assuming *B* >> *r* and *B* >> *X_T_*, the target-to-sensors distances $R_{T}\;\mathrm{and}\;R_{R}$ can be written as:

(40) $$\begin{array}{l} R_{T}=\sqrt{B^{2}+r^{2}-2rB\cos\left(\alpha -\vartheta \right)+{\left(x^{\prime }-x\right)}^{2}}≅\\ ≅B-r\cos\left(\alpha -\vartheta \right)+\frac{r^{2}}{2B}\sin^{2}\left(\alpha -\vartheta \right)+\frac{{\left(x^{\prime }-x\right)}^{2}}{2B} \end{array}$$

(41) $$R_{R}=\sqrt{r^{2}+x^{2}}$$

so that

(42) $$R_{T}+R_{R}≅B+\beta \left(\vartheta \right)r+\Delta r\left(x,r\right)+\Delta R\left(x^{\prime }-x\right),$$

where

(43) β(ϑ)=1−cos(α−ϑ),

(44) $$\Delta r\left(x,r\right)=\frac{r^{2}}{2B}\sin^{2}\left(\alpha -\vartheta \right)+\sqrt{r^{2}+x^{2}}-r,$$

(45) $$\Delta R\left(x^{\prime }-x\right)=\frac{{\left(x^{\prime }-x\right)}^{2}}{2B}.$$

By assuming that the transmitted pulse is an up-chirp with bandwidth Δf and duration τ, the PSF (after coherent demodulation of the raw signal) can be written as:

(46) $$\begin{array}{c} f_{Rx}\left(x^{\prime },x,t-t_{n},r\right)=\exp\left[-j\frac{2\pi }{\lambda }\left(B+\beta r+\Delta r+\Delta R\right)\right]\\ \exp\left[j\frac{\pi \Delta f}{\tau }{\left(t-t_{n}-\frac{B+\beta r+\Delta r+\Delta R}{c}\right)}^{2}\right]\mathrm{rect}\left[\frac{1}{\tau }\left(t-t_{n}-\frac{B+\beta r+\Delta r+\Delta R}{c}\right)\right]\\ \mathrm{rect}\left(\frac{r-r_{0}}{S_{r}}\right)\mathrm{rect}\left(\frac{x}{X_{R}}\right)\mathrm{rect}\left(\frac{x^{\prime }-x}{X_{T}}\right)W_{T}\left(\frac{x^{\prime }-x}{X_{T}}\right)W_{R}\left(\frac{x}{X_{R}}\right) \end{array}$$

Finally, by letting $r^{\prime }=\frac{c\left(t-t_{n}\right)-B}{\beta_{0}}$, with $\beta_{0}=\beta (\vartheta_{flat}\left(r_{0}\right))=\beta \left(\vartheta_{0}\right)$, the raw signal in (15) and (39) can be expressed as:

(47) $$h\left(x^{\prime },r^{\prime }\right)=\iint \tilde{\gamma }\left(x,r\right)g\left(x^{\prime }-x,\;r^{\prime }-r;x,r\right)dxdr,$$

where

(48) $$\tilde{\gamma }\left(x,r\right)=\gamma \left(x,r\right)\exp\left\{-j\frac{2\pi }{\lambda }\left[B+\beta \left(\vartheta \left(x,r\right)\right)r+\Delta r\left(x,r\right)\right]\right\}\mathrm{rect}\left(\frac{r-r_{0}}{S_{r}}\right)\mathrm{rect}\left(\frac{x}{X_{R}}\right)W_{R}\left(\frac{x}{X_{R}}\right)$$

and

(49) $$\begin{array}{c} g\left(x^{\prime }-x,\;r^{\prime }-r;x,r\right)≅\exp\left[-ja{\left(x^{\prime }-x\right)}^{2}\right]\\ \exp\left[jb{\left(r^{\prime }-r-\left(\beta_{f}-\beta_{0}\right)r-\frac{2\pi \Delta r_{f}+\lambda a{\left(x^{\prime }-x\right)}^{2}}{2\pi \beta_{0}}\right)}^{2}\right]\\ \mathrm{rect}\left[\frac{\beta_{0}}{c\tau }\left(r^{\prime }-r-\left(\beta_{f}-\beta_{0}\right)r-\frac{2\pi \Delta r_{f}+\lambda a{\left(x^{\prime }-x\right)}^{2}}{2\pi \beta_{0}}\right)\right]W\left(\frac{x^{\prime }-x}{X_{T}}\right) \end{array}$$

with

(50) $$a=\frac{\pi }{\lambda B},\;b=\frac{\beta_{0}^{2}\pi }{\lambda }\frac{\Delta f/f}{c\tau },\;W\left(\frac{x^{\prime }-x}{X_{T}}\right)=\mathrm{rect}\left(\frac{x^{\prime }-x}{X_{T}}\right)W_{T}\left(\frac{x^{\prime }-x}{X_{T}}\right),$$

and $\beta_{f}$ and $\Delta r_{f}$ are obtained from β and Δr by letting $\vartheta \left(x,r\right)≅\vartheta_{flat}\left(r\right).$

Note that in this case of one-stationary configuration both *a* and *b* are constant, but the pulse response *g* is both range and azimuth variant, due to the dependence on *r* of $\beta_{f}$ and on both *r* and *x* of $\Delta r_{f}$. Nevertheless, the bistatic raw signal of (47)–(49) still has a form similar to the one of the monostatic SAR raw signal of [15,16,17,18,19,20,25], although with different expressions for the parameters a and b. Therefore, the same asymptotic stationary-phase evaluation of its two-dimensional (2-D) Fourier Transform (FT) can be performed, thus obtaining:

(51) $$H\left(\xi ,\eta \right)=\iint \tilde{\gamma }\left(x,r\right)G\left(\xi ,\eta ;x,r\right)\exp\left(-j\xi x-j\eta r\right)dxdr,$$

where

(52) $$G\left(\xi ,\eta ;x,r\right)=\exp\left\{j\left[\frac{\xi^{2}}{4a\left(1+\frac{\eta \lambda }{2\pi \beta_{0}}\right)}-\frac{\eta^{2}}{4b}-\eta \left(\beta_{f}-\beta_{0}\right)r-\frac{\eta \;\Delta r_{f}}{\beta_{0}}\right]\right\}W\left[\frac{\xi -\xi_{DC}}{2aX_{T}}\right]\mathrm{rect}\left[\frac{\eta }{2bc\tau /\beta_{0}}\right].$$

Range variance of this TF can be handled as in the TI configuration case by letting:

(53) $$G\left(\xi ,\eta ;x,r\right)≅G_{0}\left(\xi ,\eta ;x\right)\exp\left[-j\eta \;\mu \left(x\right)\left(r-r_{0}\right)\right],$$

where $G_{0}\left(\xi ,\eta ;x\right)=G\left(\xi ,\eta ;x,r_{0}\right)$ and

(54) $$\mu \left(x\right)={\frac{\partial }{\partial r}\left[\left(\beta_{f}-\beta_{0}\right)r+\frac{\;\Delta r_{f}}{\beta_{0}}\right]|}_{r=r_{0}}≅\frac{\sin\left(\alpha -\vartheta_{0}\right)}{\tan\vartheta_{0}}-\frac{x^{2}}{2\beta_{0}r_{0}^{2}}$$

If $\Delta r_{f}\left(x,r_{0}\right)-\Delta r_{f}\left(0,r_{0}\right)≅\frac{x^{2}}{2r_{0}}\ll \beta_{0}/\left|\eta \right|$, then we can let $G_{0}\left(\xi ,\eta ;x\right)≅G_{0}\left(\xi ,\eta ;0\right)$ and $\mu \left(x\right)≅\mu \left(0\right)=\mu_{0}$, so that azimuth variance of the TF can be ignored, and (51) can be written as:

(55) $$H\left(\xi ,\eta \right)=\tilde{\Gamma }\left[\xi ,\left(1+\mu_{0}\right)\eta \right]G_{0}\left(\xi ,\eta ;0\right)$$

and, again, an extended-scene bistatic SAR raw signal simulation scheme similar to the monostatic one of [15,16,17,18,19,20] can be employed, as described in the previous subsection. This happens if the following condition is satisfied:

(56) $$\frac{X_{R}^{2}\pi \Delta f}{8r_{0}c}<\frac{\pi }{4}$$

For instance, if Δf= 30 MHz and $r_{0}=3\;\mathrm{km}$, we get *X_R_* < 250 m. If condition (56) is not satisfied, the scene must be divided into *N* azimuth slices of width *X_Rn_* satisfying (56) and of central azimuth coordinate *x_n_*. For each slice, the above-described simulation scheme can be used, with $G_{0}\left(\xi ,\eta ;x\right)≅G_{0}\left(\xi ,\eta ;x_{n}\right)$ and $\mu \left(x\right)≅\mu \left(x_{n}\right)=\mu_{n}$, so that we get:

(57) $$H_{n}\left(\xi ,\eta \right)=\tilde{\Gamma }\left[\xi ,\left(1+\mu_{n}\right)\eta \right]G_{0}\left(\xi ,\eta ;x_{n}\right)$$

and the final overall raw signal is obtained by summing up the raw signals obtained from the *N* slices.

## 4. Numerical Results and Discussion

This Section is devoted to illustrating the results of numerical simulations relevant to the two bistatic SAR configurations described in Section 3.1 and Section 3.2. In particular, for both configurations, we provide a comparison between the point-target raw signals simulated via the (exact) time domain method of Section 2 and via the (approximated) frequency-domain ones of Section 3, in order to validate the latter. Presented raw signals lie in the slow-time/fast-time plane; the slow-time duration of each raw signal corresponds to the time slot within which the look function is equal to 1, while its fast-time duration is the difference between the maximum and the minimum round trip of the transmitted pulse increased by the pulse duration.

Let us consider the TI bistatic SAR system whose parameters are listed in Table 1. Such parameters are those of a typical C-band SAR system: in particular, parameters common to all of the three following examples are based on a planned system, described in [10] and references therein. The three examples differ one from another for the considered baseline.

**Figure 5:**
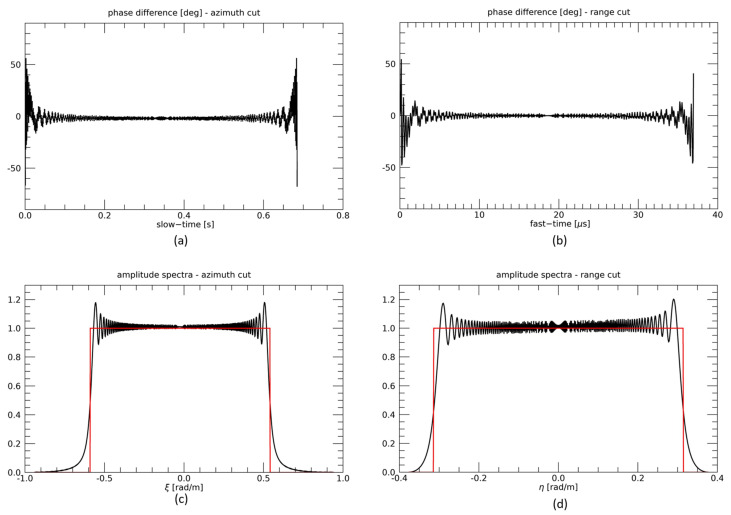
TI configuration, first example. Slow-time (**a**) and fast-time (**b**) cuts of the difference between the phases of the raw signals simulated in time and frequency domains. Slow-time (**c**) and fast-time (**d**) cuts of spectra of raw signals generated via the time (black line) and frequency (red line) domain simulators.

The first example refers to a receiver that is a part of a formation conceived for tomographic applications [28], so that we consider a large cross-track baseline (*B* = 8 km) and a rather small along track baseline (*d* = 800 m). In Figure 5a,b, we show slow-time and fast-time cuts of the difference between the phases of the raw signals simulated in time and frequency domains: the absolute value of this difference is only very few degrees, except that at the very edge of the raw signals, where however, it does not exceed about 50 degrees. Spectra of raw signals generated via the time and frequency domain simulators are compared in Figure 5c,d. A very good agreement is obtained: differences are only related to the use of the phase stationary method for the evaluation of the system TF, so that small amplitude oscillations of the signal spectrum are lost by the frequency domain simulator, but the spectrum mean amplitude and the bandwidth are perfectly reconstructed.

**Figure 6:**
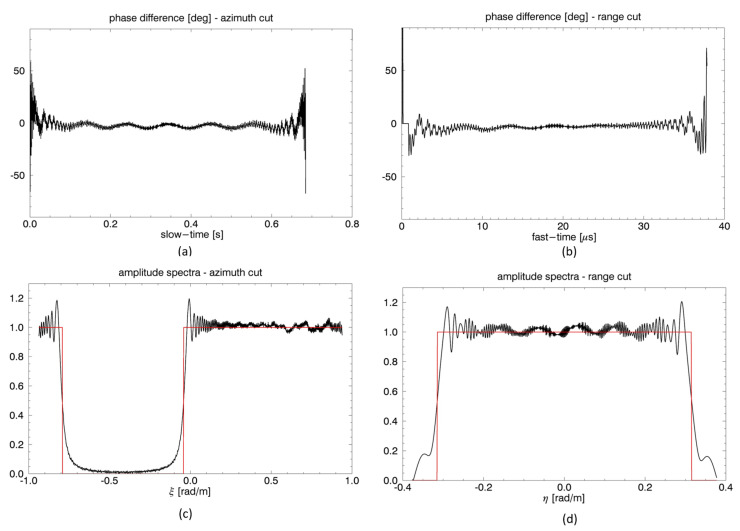
TI configuration, second example. Slow-time (**a**) and fast-time (**b**) cuts of the difference between the phases of the raw signals simulated in time and frequency domains. Slow-time (**c**) and fast-time (**d**) cuts of spectra of raw signals generated via the time (black line) and frequency (red line) domain simulators.

In the second example, we consider system parameters of a receiver belonging to an along-track FF-SAR configuration, conceived for wide-swath imaging and SNR enhancement [8,9,10]. More specifically, we refer to the system described in [10], in which the receivers’ formation follows the transmitter, used as an illuminator of opportunity, at large distance (several tens km), to ensure that the functionalities of the transmitting platform (e.g., telemetry, tracking, and command) remain unaffected by the presence of the receiving formation. Therefore, we consider a very large along-track baseline (*d* = 50 km), and a very small cross-track one (*B* = 20 m), that in real missions can be present owing to inaccuracy of the relative orbit control but also as a nominal condition to minimize the collision risk [10]. Results of this numerical experiment are reported in Figure 6 according to the same format of Figure 5. The phase difference between results of time-domain and frequency domain simulation is slightly larger than the one of the previous example, but it is still limited to less than about 5 degrees, except that at the very edge of the raw signals, where again it does not exceed about 50 degrees. In this case, it is interesting to note that the frequency-domain simulator correctly accounts for the spectral shift induced by the very large Doppler centroid related to the very large along-track baseline, as illustrated in Figure 6c.

**Figure 7:**
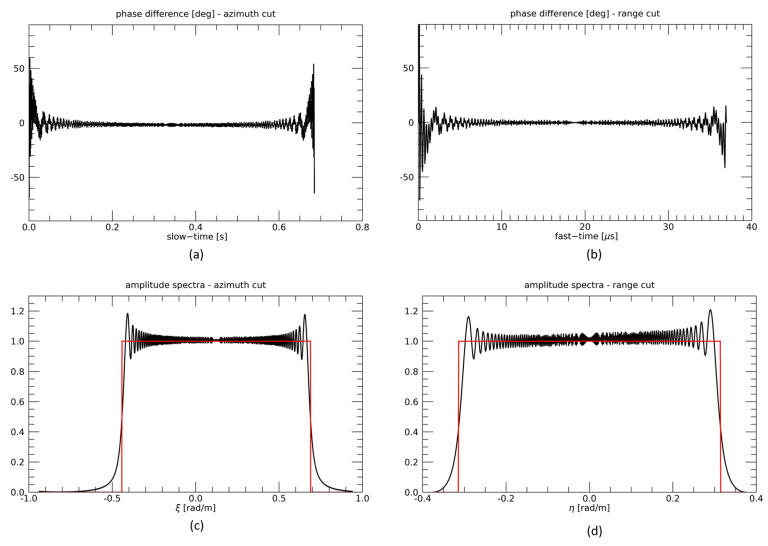
TI configuration, third example. Slow-time (**a**) and fast-time (**b**) cuts of the difference between the phases of the raw signals simulated in time and frequency domains. Slow-time (**c**) and fast-time (**d**) cuts of spectra of raw signals generated via the time (black line) and frequency (red line) domain simulators.

In order to fully show the potentiality of the presented frequency-domain simulation, we consider a third example in which both cross- and along-track baselines are large (*B* = 12 km, *d* = 13 km). Results in this case, see Figure 7, are similar to those of the first example, thus confirming that the frequency domain simulation scheme can account for large values of all baseline components.

**Figure 8:**
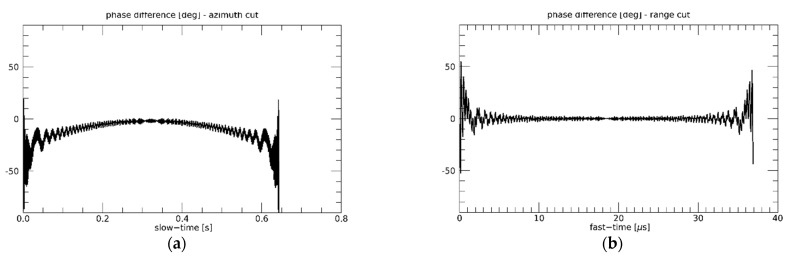
One-stationary configuration. Slow-time (**a**) and fast-time (**b**) cuts of the difference between the phases of the raw signals simulated in time and frequency domains.

Finally, let us consider the one-stationary, space-surface bistatic SAR system whose parameters are listed in Table 2. Additionally, for this configuration, the phase error of the approximated frequency-domain simulation approach is very small, although a slight (quadratic) increase can be noticed moving from the center to the edge of the raw signal, see Figure 8. However, excluding the raw signal edge, it does not exceed about 10 degrees. 

## 5. Conclusions

We have shown that the transfer function of translational-invariant and one-stationary bistatic SAR systems can be formulated in a way formally very similar to the one of usual monostatic SAR systems. This allows us to devise computationally efficient simulation schemes, suitable for extended scene simulation and working in the frequency domain, that are very similar to the available ones, employed for monostatic SAR simulation. Therefore, the latter can be easily adapted to these bistatic cases. 

A comparison has been performed between the point-target raw signals simulated via the exact time-domain simulation method (here reformulated in an original form) and via the proposed approximated frequency-domain ones. Results confirm viability of the proposed frequency-domain simulation schemes. In fact, the phase difference between raw signals simulated with the two approaches turns out to be in all cases smaller (and often much smaller) than about 10 degrees, except that at the very edge of the raw signals, where however it does not exceed about 50 degrees. 

We finally want to explicitly emphasize that, while already available bistatic systems, see e.g., [4], are basically single-pass interferometric SARs (i.e., the baseline is very small with respect to the sensor-to-ground distance), our simulator can account for very large along-track (TI case) or cross-track (one-stationary case) baselines, so being able to help planning next-generation SAR bistatic systems implementing FF-SAR and space-surface configurations.

## Figures and Tables

**Table 1:** System parameters of the TI bistatic SAR configuration considered in the simulations.

Parameter	Measurement Unit	Value
Carrier frequency *f*	GHz	5.1
Chirp bandwidth Δ*f*	MHz	15
Chirp duration *τ*	μs	37
Sampling frequency *f_s_*	MHz	18
Pulse repetition frequency PRF	kHz	2000
Transmitting and receiving antennas sizes (cross-track × along-track)	m × m	1 × 11.1
Polarization		VV
Transmitter and receiver velocity *v*	m/s	6691
Transmitter platform height *h_T_*	km	775
Transmitter look angle *ϑ*_0*T*_	deg	30
Cross-track baseline *B*	m	First example: 8000 Second example: 20 Third example: 12,000
Cross-track baseline angle *α*	deg	120
Along-track transmitter baseline *d_T_*	m	First example: 500 Second example: 0 Third example: 6000
Along-track receiver baseline *d_R_*	m	First example: 300 Second example: 50,000 Third example: 7000
Target position (*x*, *y*, *z*)	m	(0, 433,000, 0)

**Table 2:** System parameters of the one-stationary bistatic SAR configuration considered in the simulations.

Parameter	Measurement Unit	Value
Carrier frequency *f*	GHz	5.3
Chirp bandwidth Δ*f*	MHz	15
Chirp duration *τ*	μs	37
Sampling frequency *f_s_*	MHz	18
Pulse repetition frequency PRF	kHz	1679
Transmitting antenna size (cross-track × along-track)	m × m	1 × 11.1
Receiving antenna size (cross-track × along-track)	m × m	0.1 × 0.1
Polarization		VV
Transmitter velocity *v*	m/s	6691
Transmitter platform height *h_T_*	km	775
Receiver platform height *h_R_*	km	1.5
Receiver look angle *ϑ*_0*R*_	deg	20
Cross-track ground-projected baseline *B*sin*α*	km	337
Target position (*x*, *y*, *z*)	m	(0, 546, 0)

## Data Availability

Not applicable.
